# Identifying the Complexity of Multiple Risk Factors for Obesity Among Urban Latinas

**DOI:** 10.1007/s10903-016-0433-z

**Published:** 2016-05-25

**Authors:** Ruth M. Masterson Creber, Elaine Fleck, Jianfang Liu, Gloria Rothenberg, Beatriz Ryan, Suzanne Bakken

**Affiliations:** 1 School of Nursing, Columbia University, 617 W 168th St, New York, NY 10032 USA; 2 Columbia University Medical Center/New York Presbyterian Hospital, 622 W 168th St, New York, NY 10032 USA; 3 Vanderbilt University, 2201 West End Ave, Nashville, TN 37235 USA; 4 The Value Institute at New York Presbyterian Hospital, 622 W 168th St, New York, NY 10032 USA

**Keywords:** Hispanics, Latinas, Obesity, Overweight, Hypertension, Diabetes mellitus, Comorbidity

## Abstract

The prevalence of obesity is rising rapidly among Hispanics/Latinas. We evaluated the prevalence of being obese or overweight and associated risk factors among 630 low-income, Latina women from ambulatory care clinics in Upper Manhattan. Overall, 37 % of the sample was overweight and 41 % of the sample was obese, and yet, almost half of women who are overweight considered their weight “just about right.” After adjusting for socio-demographic, behavioral, and biological risk factors, being obese was strongly associated with having hypertension [relative risk ratio (RRR) 3.93, 1.75–8.82], pre-hypertension (RRR 2.59, 1.43–4.67), diabetes (RRR 2.50, 1.21–5.14) and moderate/moderately severe/severe depression (RRR 2.09, 1.03–4.26). Women who reported that finding time was a barrier to physical activity were also more likely to be obese (RRR 1.78, 1.04–3.02). Chronic financial stress was associated with lower risk of being overweight (RRR 0.47, 0.28–0.79) or obese (RRR 0.51, 0.31–0.86), as well as eating out at restaurants (RRR 0.75, 0.62–0.89). Opportunities for intervention relate to understanding cultural factors around perceptions of weight and helping women find the time for physical activity.

## Introduction

Hispanics/Latinos are the fastest growing ethnic group in the U.S. Currently Hispanics/Latinos comprise almost half of the U.S. immigrant population and by 2060 are estimated to represent 29 % of the U.S. population [1]. Dominicans are the fifth largest subgroup of Hispanics/Latinos living in the U.S. [2] and nearly half reside in New York City [3, 4].

Nationally, the increase in the number of obese adults appears to be leveling off; however, the prevalence of obesity is not declining [5]. Disparities in the prevalence of obesity are actually increasing among Blacks and Hispanics/Latinos [5–7]. Based on 2011–2012 NHANES data, the age-adjusted prevalence of obesity among non-Hispanic white women was estimated to be 32.8 % compared to 44.4 % of all Hispanic/Latina women [8]. Major differences in health outcomes and health related habits exist among specific nationalities of Hispanics/Latinos [9], due to several factors such as cultural differences in dietary habits, prevalence of depressive symptoms, and levels of adherence to medical recommendations [10]. The Hispanic Community Health Study/Study of Latinos, which reports on the prevalence of cardiovascular risk factors, including obesity among different ethnic groups of Latinas, indicates that Puerto Rican and Dominican women have the highest prevalence of obesity (51.4 and 42.5 % respectively) in the U.S. [9, 10]. Obesity is important to prevent because it increases risk for cardiovascular disease in women by 64 % [5], increases risk of hypertension, which is a leading cause of death and disability [11] especially in minority populations [12].

The Centers for Disease Control and Prevention identified the reduction of obesity amongst Hispanics/Latinos as such a high priority that they published a tool kit titled, “Health Equity Resource Toolkit for State Practitioners Addressing Obesity Disparities,” to create policy, systems, and environmental changes to reduce obesity disparities and achieve health equity [13]. In addition, there have been a number of interventions aimed at reducing obesity in this population, including a 2-year randomized controlled trial comparing usual care with a case-management and community health care worker weight loss intervention [14] in a sample comparable to the Washington Heights and Inwood Informatics Infrastructure for Comparative Effectiveness Research (WICER) study. Perez and colleagues also reported the results of a systematic review of 22 studies on obesity treatment interventions conducted in the U.S. for overweight or obese Latino adults (1990–2010) [15]. Understanding risk factors for being overweight or obese provides an essential foundation for designing relevant interventions for Latinas.

This work is a secondary analysis of WICER study data from participants who were recruited directly from four NewYork-Presbyterian Hospital-Columbia University Medical Center Ambulatory Care Network clinics. The objective of this paper is to identify risk factors for being overweight or obese among Latinas.

## Methods

### Participants

Data were collected between 2010–2013 in the New York City neighborhoods of Washington Heights and Inwood (zip codes 10031, 10032, 10033, 10034, and 10040). These two neighborhoods have approximately 280,000 people, most of whom are Latino and foreign born, mainly from the Dominican Republic (71 %). In this community, less than 50 % are proficient in English. A convenience sampling methodology was employed for participant recruitment from four ambulatory care network clinics.

Bilingual research assistants introduced the study to patients while they were waiting in the clinic reception area. If patients were interested in the study they were taken to a private exam room where they completed the informed consent in English or Spanish. After signing informed consent, patients were interviewed in the language of their choice and had their weight, height and blood pressure measured using standardized techniques and equipment. On average, the questionnaires and measurements took about 45–60 min for participants to complete. All study procedures were approved by the Columbia University Institutional Review Board. Participants were compensated with a $25 grocery voucher for their time.

### Measures

The WICER survey included comprehensive sociocultural questions pertaining to demographic information, socio-demographic and lifestyle factors, acculturation, health and healthcare behavior, blood pressure and body size, general health, mental health, physical activity and diet, sleep and energy, social relations, psychosocial and cognitive processes, the neighborhood environment, alcohol intake, smoking history and health literacy. Detailed information on the questionnaire content, outcome and covariate measurements have been published [16] and are summarized here.

### Outcome Measurement

Body mass index (BMI) was calculated as weight in kilograms divided by height in meters squared and rounded to the nearest tenth. Following current recommendations, healthy weight was defined as a BMI of 18.5–24.9, overweight was defined as a BMI of 25.0–29.9 and obesity as a BMI of 30.0 or higher [17].

### Covariate Measurement

Socio-demographic and clinical factors were self-reported and blood pressure was measured objectively. Participants were asked a number of questions about health behaviors, including questions about physical activity and exercise that were adapted from the New York City Health and Nutrition Examination Survey and the Behavioral Risk Factor Surveillance System [18]. Sample physical activity questions included how many days they walked or bicycled to and from work or school and how many days of the month they performed vigorous, moderate and mild activities. Participants also responded to five questions taken from the environmental questions of the Influences on Physical Activity Instrument [19] about barriers to physical activity, including finding the time, affording the cost, and finding a place for physical activity. Dietary questions included questions about fruit, vegetable, and soda consumption, as well as the number of times per week that participants ate out at restaurants.

In this study, depressive symptoms were measured using a modified Patient Health Questionnaire-9 (PHQ-9) [20]. In the modified PHQ-9, participants were asked if they had experienced a period of at least 2 weeks over the last 30 days in which they were bothered by specific symptoms of depression. The rationale for the modified timeframe was to align with other 30-day measures in the WICER survey. The modified PHQ-9 had high internal reliability (Cronbach’s alpha is 0.921) in the study sample. Traditionally, PHQ-9 scores (0–27) have been divided into five categories; however, for the purposes of this study we collapsed them into three categories: none/minimal (0–4), mild (5–9) or moderate/moderately severe/severe (10–27).

Anxiety [21], depression [21] and sleep disturbance [22] were measured using Patient Reported Outcome Measurement Information System (PROMIS) short forms. Self-reported general health was measured using the Center for Disease Control Health Related Quality Of Life-4 module [23], which categorized self-reported health as excellent, very good, good, fair or poor. We recoded self-reported health into two categories: good/very good/excellent versus fair/poor (as recommended in previous publications) [24]. Chronic stress was measured using the chronic stress scale [25] in which participants were asked to identify ongoing problems across five domains [health (self), health (loved one), job, relationship and financial problems] over the last 6 months. A total score was calculated by summing the total number of items to which a “yes” response was given (range 0–5). Each domain of stress was also reported individually, as in other publications [26, 27], in order to differentiate which burdens women were experiencing and how specific chronic stresses may confer differential risk of being overweight or obese.

Blood pressure was measured systematically for all participants using an automated sphygmomanometer (BpTRU Model BPM-200). Participants sat in a chair with feet on the floor, back and arm supported, with the cuff at the level of the heart. Three measurements were taken on an automatic cycle of 1 or 2 min, as recommended by the manufacturer and the American Heart Association. The systolic blood pressure measure used in this analysis was an average of the 2nd and 3rd measurements of the systolic blood pressure values.

### Analysis

Descriptive statistics were used to calculate frequencies with percentages and means with standard deviations for the total sample and by BMI categories. The primary outcome of the study was being overweight or obese and the reference group was normal weight. Multinomial logistic regression models were used to examine variables that are associated with being overweight or obese. Covariate selection started with a priori factors consistent with both the individual and interpersonal factors identified in the social ecological model for addressing obesity disparities [28, 29], including age [5], education [30], Medicaid as a proxy measure of socioeconomic status [31], and diabetes [32]. Second, bivariate associations between the independent variables and the dependent variable were quantified using ANOVA for continuous variables and Chi square for categorical variables. If the *p* value was less than 0.20 from the bivariate associations then the potential independent variable was considered for inclusion in the final model [33]. A robust model comparison approach [34] was applied using a stepwise model building process starting with a basic model and comparing it with a model in which one or more variables were added. The decision to include or not to include an independent variable in the final model was made based on the differences in Akaike’s Information Criterion with smaller values indicating better fit. The final model included the following 12 variables: age, education, Medicaid insurance, diabetes, systolic blood pressure, depression, self-reported health, number of times going to a restaurant per week, time as a barrier to physical activity, hours spent watching television, chronic financial and personal health stress. Estimates of relative risk ratios (RRR) and confidence intervals of the RRR are reported. All analyses were done using StataSE 13.1 (College Station, TX) and Version 9.3 of the SAS System for Windows (SAS Institute 2011).

## Results

A total of 630 participants were included in this sample (mean age 49 years) (Table 1). All of the participants self-identified as Latina, 32 % had less than 8th grade education and 81 % were on Medicaid. The majority of the participants were either overweight (37 %) or obese (41 %). About half of the women who were overweight and 14 % of the women who were obese considered their weight “just about right.” Over half of the participants reported poor perceived health and 26 % of obese women reported having moderate/moderately severe/severe depression over a period of 30 days based on the modified PHQ-9. Almost a quarter of the sample had self-reported diabetes and the prevalence was highest among obese women (31 %). Thirty percent of the sample had prehypertension [systolic blood pressure (SBP) 120–140 mmHg] [35] and 19.2 % had hypertension (SBP > 140 mmHg) [35].

**Table 1:** Baseline socio-demographic, lifestyle and clinical characteristics of Latino females by body mass index (n = 630)

	Body mass index [mean (SD) or N (%)]				
Variables	Overall N = 630 (%), Mean (SD)	Normal N = 142 (22.5)	Overweight N = 230 (36.5)	Obese N = 258 (41.0)	*p* value
Age	48.5 (±16.7)	43.5 (±17.9)	50.2 (±16.9)	49.7 (±15.4)	**<0.001**
Married/partnered	227 (36.2)	42 (30.0)	91 (39.6)	94 (36.6)	0.368
Country of origin					
Dominican republic	457 (72.8)	99 (21.7)	178 (39.0)	180 (39.4)	0.167
Cuba	8 (1.3)	3 (37.5)	2 (25)	3 (37.5)	
Mexico	25 (4.0)	4 (16.0)	11 (44.0)	10 (40.0)	
Ecuador	18 (2.9)	1 (5.6)	9 (50.0)	8 (44.4)	
Puerto Rico	8 (1.3)	1 (12.5)	2 (25)	5 (62.5)	
United States	96 (15.3)	28 (29.2)	22 (22.9)	46 (47.9)	
Hispanic, Latino or Spanish origin	630 (100)	142 (100)	230 (100)	258 (100)	NA
Race					0.863
White of Caucasian	28 (4.5)	7 (5.0)	7 (3.1)	14 (5.4)	
Black or African American	20 (3.2)	5 (3.6)	8 (3.6)	7 (2.7)	
Other race	507 (81.5)	112 (80.6)	188 (83.6)	205 (79.5)	
Don’t know/refused	14 (10.0)	22 (9.74)	31 (12.1)	67 (10.8)	
Education					**0.051**
Eighth grade or less/never	202 (32.2)	33 (23.4)	75 (32.6)	94 (36.4)	
Some High School	94 (15.0)	21 (14.8)	37 (16.1)	36 (14.0)	
High School/some college	225 (35.9)	53 (37.6)	78 (34.1)	94 (36.7)	
College/MA/PhD	106 (16.9)	34 (24.1)	39 (17.0)	33 (12.8)	
Medicaid	506 (80.8)	118 (83.7)	190 (83.7)	198 (76.7)	0.094
Poor perceived health	323 (51.6)	52 (36.9)	117 (51.5)	154 (59.7)	**<0.001**
Hours per week of physical activity	13.3 (±18.5)	15.7 (±21.9)	13.4 (±17.7)	11.9 (±16.4)	0.147
Physical activity barriers					
Accessible places	176 (28.2)	27 (19.3)	70 (30.7)	79 (30.9)	**0.029**
Cost of physical activity	301 (48.2)	54 (38.3)	118 (51.8)	129 (50.4)	**0.028**
Hard to find time	202 (32.3)	36 (25.5)	66 (28.8)	100 (39.0)	**0.008**
Cannot make time	517 (82.9)	123 (87.9)	185 (81.1)	209 (81.6)	0.202
Self-perceived weight					**<0.001**
Underweight	29 (4.6)	18 (12.9)	10 (4.4)	1 (0.39)	
Just about right	263 (42.0)	114 (81.4)	113 (49.1)	36 (14.1)	
Overweight	328 (52.4)	8 (5.7)	103 (44.8)	217 (84.8)	
Eating out at a restaurant per week	0.9 (±2.7)	1.3 (±2.1)	0.64 (±1.1)	0.82 (±1.3)	**<0.001**
Sodas per week	1.4 (±3.6)	1.3 (±3.5)	1.5 (±3.0)	1.4 (±4.0)	0.933
Fruit per week	5.6 (±4.6)	5.7 (±4.2)	5.8 (±4.3)	5.4 (±4.9)	0.562
Dark vegetables per week	3.2 (±4.8)	2.7 (±2.5)	3.2 (±3.9)	3.5 (±6.3)	0.277
Sugary fruit drinks per week	2.1 (±4.4)	3.3 (±4.6)	3.5 (±4.1)	3.0 (±4.7)	0.650
Watching TV > 1 h per day	419 (66.9)	85 (60.3)	145 (63.0)	189 (74.1)	**0.006**
Diabetes	137 (21.8)	167(12.0)	41 (17.9)	79 (30.6)	**<0.001**
Systolic blood pressure					**<0.001**
SBP <120 mmHG (normotensive)	317 (50.8)	99 (69.7)	125 (54.6)	93 (36.7)	
SBP 120–140 mmHG (pre-hypertensive)	187 (30.0)	31 (21.8)	61 (26.6)	95 (37.6)	
SBP > 140 mmHG (hypertensive)	120 (19.2)	12 (8.5)	43 (18.8)	65 (25.7)	
Depression (PHQ-9)					**0.018**
None/minimal (0–4)	400 (64.3)	101 (72.7)	152 (66.1)	147 (58.1)	
Mild (5–9)	95 (15.3)	21 (15.1)	34 (14.8)	40 (15.8)	
Moderate/moderately severe/severe (10–27)	127 (20.4)	17 (12.2)	44 (19.1)	66 (26.1)	
PROMIS measures^a^					
Anxiety	50.0 (±11.3)	50.1 (±11.3)	49.0 (±11.1)	50.8 (±11.5)	0.227
Depression	48.3 (±10.2)	47.3 (±9.2)	48.0 (±10.4)	49.1 (±10.5)	0.218
Sleep disturbance	52.2 (±3.4)	51.9 (±3.8)	52.1 (±3.3)	52.5 (±3.2)	0.207
Chronic stress burden					
Personal health problems	172 (27.4)	24 (17.0)	66 (28.8)	82 (31.9)	**0.005**
Health problems with someone close	206 (32.9)	39 (27.9)	79 (34.5)	88 (34.2)	0.352
Job difficulties	76 (12.0)	17 (12.1)	28 (12.2)	31 (12.0)	0.999
Financial problems	207 (33.1)	54 (38.6)	63 (27.8)	90 (34.9)	0.075
Relational difficulties	62 (9.9)	12 (8.6)	23 (10.0)	27 (10.5)	0.841

Bold value indicates *p* < 0.05

*SD* standard deviation, *N* number, *SBP* systolic blood pressure, *PHQ-9* Patient Health Questionnaire-9

^a^PROMIS measures are reported as T-scores

The barriers to physical activity amongst obese women included finding the time (39 %), costs of physical activity (50 %), and lack of accessible places (31 %). The majority of participants (67 %) also spent an hour or more watching television per day and this was highest among obese participants (74 %). On average, participants reported eating out at restaurants less than once per week.

The unadjusted RRR are presented in Table 2 and the adjusted RRR are presented in Table 3. In the final multiple multinomial logistic regression model, obesity was strongly associated with chronic comorbid conditions. Women with hypertension (RRR 3.93; 1.75–8.82) or pre-hypertension (RRR 2.59; 1.43–4.67) were much more likely to be obese compared to normotensive women. Likewise, women with diabetes (RRR 2.50; 95 % CI 1.21–5.14) were more likely to be obese. Women with moderate or greater depression were more likely to be obese (RRR 2.09; 1.03–4.26) compared to women who reported none/minimal depression using the PHQ-9. Women who reported that finding time was a barrier to physical activity were also more likely to be obese (RRR 1.78; 1.04–3.02). The relative risk of being overweight was 25 % less for each additional time that women ate out at a restaurant per week [95 % CI 0.62–0.89]. Chronic financial stress was associated with a lower risk of being overweight (RRR 0.47, 0.28–0.79) or obese (RRR 0.51, 0.31–0.86) (Table 3).

**Table 2:** Unadjusted relative risk ratios of predictors of being overweight or obese compared to normal weight

Variables	RRR	95 % Confidence interval
*Associations with being overweight*		
Age	1.02	1.01–1.03**
Education: eighth grade higher	0.66	0.40–1.09
Medicaid	1.38	0.79–2.41
Diabetes	1.92	0.98–3.79
Systolic blood pressure		
SBP 120–140 mmHG (pre-hypertensive)	1.50	0.88–2.56
SBP > 140 mmHG (hypertensive)	2.69	1.33–5.44**
Depression (measured with the PHQ-9)		
Mild	1.21	0.64–2.28
Moderate/moderately severe/severe	1.64	0.85–3.16
Poor self-reported health	1.74	1.11–2.73*
Eating out at restaurants per week	0.71	0.60–0.83**
Physical activity barrier (time)	1.18	0.71–1.95
TV > 1 h per day	1.15	0.73–1.82
Chronic financial stress	0.59	0.37–0.93*
Chronic stress about personal health	0.54	0.31–0.94*
*Associations with being obese*		
Age	1.02	1.01–1.04**
Education: eighth grade higher	0.51	0.31–0.83**
Medicaid	0.89	0.53–1.49
Diabetes	4.12	2.18–7.79**
Systolic blood pressure		
SBP 120–140 mmHG (pre-hypertensive)	3.18	1.90–5.32**
SBP > 140 mmHG (hypertensive)	5.15	2.59–10.26**
Depression (measured with the PHQ-9)		
Mild depression	1.34	0.72–2.52
Moderate/severe	2.94	1.58–5.47**
Poor self-reported health	2.42	1.55–3.76**
Eating out at restaurants per week	0.81	0.71–0.93**
Physical activity barrier (time)	1.91	1.18–3.10**
TV > 1 h per day	1.80	1.14–2.86*
Chronic financial stress	0.77	0.50–1.21
Chronic stress about personal health	0.46	0.27–0.79**

*PHQ-9* Patient Health Questionnaire-9

* Statistically significant at 0.05 level

** Statistically significant at 0.01 level

**Table 3:** Adjusted relative risk ratios of predictors of being overweight or obese compared to normal weight from the final multinomial logistic regression model

Variables	RRR	95 % Confidence interval
*Associations with being overweight*		
Age	1.00	0.98–1.02
Education: eighth grade higher	1.12	0.61–2.04
Medicaid	1.10	0.61–2.00
Diabetes	1.13	0.53–2.38
Systolic blood pressure		
SBP 120–140 mmHG (pre-hypertensive)	1.15	0.63–2.09
SBP > 140 mmHG (hypertensive)	2.02	0.90–4.54
Depression (measured with PHQ-9)		
Mild	1.05	0.53–2.06
Moderate/moderately severe/severe	1.34	0.64–2.80
Poor self-reported health	1.23	0.71–2.12
Eating out at restaurants per week	0.75	0.62–0.89**
Physical activity barrier (time)	1.33	0.77–2.29
TV > 1 h per day	0.94	0.58–1.53
Chronic financial stress	0.47	0.28–0.79**
Chronic stress about personal health	0.56	0.30–1.05
*Associations with being obese*		
Age	0.99	0.97–1.00
Education: eighth grade higher	0.84	0.46–1.53
Medicaid	0.73	0.41–1.31
Diabetes	2.50	1.21–5.14*
Systolic blood pressure		
SBP 120–140 mmHG (pre-hypertensive)	2.59	1.43–4.67**
SBP > 140 mmHG (hypertensive)	3.93	1.75–8.82**
Depression (measured with PHQ-9)		
Mild	1.07	0.54–2.13
Moderate/moderately severe/severe	2.09	1.03–4.26*
Poor self-reported health	1.24	0.72–2.15
Eating out at restaurants per week	0.87	0.75–1.03
Physical activity barrier (time)	1.78	1.04–3.02*
TV > 1 h per day	1.35	0.82–2.23
Chronic financial stress	0.51	0.31–0.86*
Chronic stress about personal health	0.61	0.32–1.14

*PHQ-9* Patient Health Questionnaire-9

* Statistically significant at 0.05 level

** Statistically significant at 0.01 level

## Discussion

The major findings of this study are that being overweight or obese is highly prevalent in this urban community of Latinas. Surprisingly, Latinas who are under chronic financial stress, as well as women who ate out at restaurants are less likely to be overweight. On the other hand, women who reported time as a barrier to doing physical activity are more likely to be obese. As expected, chronic co-morbid conditions (hypertension, diabetes, depression) are strongly associated with being obese among low-income, urban, predominantly Dominican women.

The finding that chronic financial stress was associated with lower risk of being overweight and obese at first may seem counter-intuitive. There is a strong assumption that stressful experiences trigger emotional eating and reduces the ability to practice positive health behaviors such as healthful eating and physical activity [36]. Another assumption is that people are more likely to consume cheap, high-calorie food in times of stress. However, the relationship between different types of stress and cardiovascular risk factors is far more nuanced. In the Hispanic Community Health Study/Study of Latinos Sociocultural Ancillary Study, investigators found chronic stress to be associated with diabetes and hypertension and yet they also reported that people who experienced more traumatic life events had a lower prevalence of diabetes and hypertension [37]. It is possible that people who successfully manage stress develop adaptive strategies for coping with future stressors that protect against obesity [37]. It is also possible that women in this sample could not afford to overeat. The relationship between different types of stress and obesity needs to be explored further in follow-up studies.

One of the more surprising findings from the study was that participants who eat out more at restaurants were less likely to be overweight. There are a few plausible explanations for this finding. First, within the Dominican population in New York City, being able to afford to eat out may be a surrogate indicator of higher socioeconomic status, which is consistent with the relationship between higher socioeconomic status and lower risk of being obese [38, 39]. Secondly, these findings could be due to nuances in how eating out at restaurants is defined. Duffrey and colleagues reported independent associations of restaurant food and fast food intake with BMI and found that fast food was positively associated with BMI, but not restaurant food consumption [40]. In this study, we could not differentiate whether participants were eating out at fast food establishments or restaurants. These results also need to be interpreted in the context of the distinct Washington Heights and Inwood neighborhoods, where there are many local restaurants, which may offer healthier options that what may be prepared at home. A limitation of the study is that at-home food choices were not adjusted for, which is recommended when evaluating the association between eating out at restaurants and obesity [41].

Women who reported that finding time was a barrier to physical activity were more likely to be obese. The literature is mixed on the impact that the barrier of time has on women being able to participate in physical activity with some studies reporting time as a barrier to physical activity [42] and others reporting that it is not [43]. In this study it was interesting to note that obese women had the highest proportion of watching TV for over an hour each day.

Consistent with results in other studies of Hispanics/Latinos, both hypertension [44] and diabetes [32, 45, 46] are strongly associated with obesity. According to a study by Bermudez and colleagues that included elderly Dominican women, 40 % of the sample was obese and the prevalence of diabetes was 43 and 41 % in the overweight and obese categories [7]. The prevalence of diabetes was higher in the Bermudez study [45] compared to this study, but that is most likely due to the mean age being 20 years older than in this study sample. In addition, being overweight and obese is a precursor to pre-diabetes and metabolic syndrome. Consistent with this study, results from a large meta-analysis report the pooled relative risk for incident diabetes were 1.87 per standard deviation of body mass index [32].

In this study, there was a positive relationship between obesity and moderate or greater depression (measured by the modified PHQ-9), consistent with other studies [47–56]. The major limitation of the previous literature on the association between obesity and depression is that sample sizes have typically not been large enough to evaluate the relationship within specific racial/ethnic groups. When the association has been examined more thoroughly within racial/ethnic minorities, the association has been inconclusive [48, 51, 53]. However, there has been growing recognition that nativity status is an important indicator in BMI and mental health outcomes among racial/ethnic groups [57]. U.S. born adults have a higher prevalence of obesity than foreign-born adults [58]. Aspects of nativity (i.e., family burden, cultural conflict, perceived neighborhood safety) are associated with depression [59]. In a study by Gavin and colleagues which examined the association between obesity and 12-month prevalence of major depression disorder [57], they found that results varied widely according to racial/ethnic status and nativity. Among Latinas, those who were obese were not more likely to have major depressive disorder, compared to non-Hispanic white women [57]. One of the differences between our study and the Gavin study was how depression was measured. In the Gavin study, the World Mental Health Initiative version of the Composite International Diagnostic Interview was used [60]. The purpose of the World Mental Health Initiative version of the Composite International Diagnostic Interview [60] is to diagnose depression, as such, it includes 40 sections that focus on diagnosis, functioning, treatment, risk factors, socio-demographic correlates and methodological factors, all of which take an average of 2 h to administer in most population samples [60]. In addition, the Gavin study used self-reported height and weight, which is likely to underestimate weight and overestimate height, thus providing an underestimate of the true prevalence of obesity in the sample [61]. This could explain the differences in the prevalence of obesity between the Gavin study, which was 31.3 % [57] compared to 41.0 % in our study. Overall, there were substantial differences in the measurement of both depression and obesity that could help to explain the contrasting findings between the two studies.

### Study Strengths/Limitations

One of the strengths of this study is that it includes a large sample of Dominican women, second only to the Hispanic Community Health Study/Study of Latinos [44]. This study also employed bilingual research coordinators to collect all study data and to make sure that patients with very low health literacy were able to fully participate in the study. In addition, all measures with the exception of the modified PHQ-9 had been previously validated with Spanish-speaking populations. Overall, the strength of this study is that it addresses a very relevant topic given the high rates of obesity in this community.

Study limitations include the fact that a convenience sampling methodology was used so the results are not generalizable outside of the clinic population. Another limitation is that diabetes was not measured with an objective measure, such as hemoglobin A1C, but instead with self-report. This is likely to underestimate the true prevalence of diabetes in the sample. Another limitation is that we could not adequately adjust for socioeconomic status. The combination of education and insurance status provided a proxy measure, but no specific questions were asked of participants that included any information related to income or self-perceived financial status.

## Conclusions

In conclusion, these study findings provide support that the prevalence of obesity continues to be a public health problem for Latinas living in New York City. Based on the results of this study, there is an urgent need for local and culturally contextualized interventions to address the complex risk factors that impact being overweight and obese, including the nutrition of at-home cooking, specific physical activity barriers, such as finding time, and perceptions of healthy weight for reducing the risk of long term cardiovascular disease.
